# Subchondral Bone Plate Changes More Rapidly than Trabecular Bone in Osteoarthritis

**DOI:** 10.3390/ijms17091496

**Published:** 2016-09-07

**Authors:** Zaitunnatakhin Zamli, Kate Robson Brown, Mohammed Sharif

**Affiliations:** 1 Centre for Comparative and Clinical Anatomy, University of Bristol, Bristol BS2 8EJ, UK; zzaitun@gmail.com; 2 Imaging Laboratory, Department of Archaeology and Anthropology, University of Bristol, Bristol BS8 1UU, UK; kate.robson-brown@bristol.ac.uk; 3 School of Clinical Sciences, University of Bristol, Musculoskeletal Research Unit, Learning and Research Building, Southmead Hospital, Bristol BS10 5NB, UK

**Keywords:** osteoarthritis, bone remodelling, bone mineral density, animal models, micro-computed tomography scanning

## Abstract

Osteoarthritis (OA) is the most common joint disorder, characterised by focal loss of cartilage and increased subchondral bone remodelling at early OA stages of the disease. We have investigated the temporal and the spatial relationship between bone remodelling in subchondral bone plate (Sbp) and trabecular bone (Tb) in Dunkin Hartley (DH, develop OA early) and the Bristol Strain 2 (BS2, control which develop OA late) guinea pigs. Right tibias were dissected from six male animals of each strain, at 10, 16, 24 and 30 weeks of age. Micro-computed tomography was used to quantify the growth plate thickness (GpTh), subchondral bone plate thickness (SbpTh) and trabecular bone thickness (TbTh), and bone mineral density (BMD) in both Sbp and Tb. The rate of change was calculated for 10–16 weeks, 16–24 weeks and 24–30 weeks. The rate of changes in Sbp and Tb thickness at the earliest time interval (10–16 weeks) were significantly greater in DH guinea pigs than in the growth-matched control strain (BS2). The magnitude of these differences was greater in the medial side than the lateral side (DH: 22.7 and 14.75 µm/week, BS2: 5.63 and 6.67 µm/week, respectively). Similarly, changes in the BMD at the earliest time interval was greater in the DH strain than the BS2, again more pronounced in the disease prone medial compartment (DH: 0.0698 and 0.0372 g/cm^3^/week, BS2: 0.00457 and 0.00772 g/cm^3^/week, respectively). These changes observed preceded microscopic and cellular signs of disease as previously reported. The rapid early changes in SbpTh, TbTh, Sbp BMD and Tb BMD in the disease prone DH guinea pigs compared with the BS2 control strain suggest a link to early OA pathology. This is corroborated by the greater relative changes in subchondral bone in the medial compared with the lateral compartment.

## 1. Introduction

Osteoarthritis (OA) is the most common joint disease and is recognised as one of the leading cause of pain and disability among the elderly. The disease is characterised by bone remodelling, cartilage degradation and variable inflammation of the synovium. These processes have been widely investigated and both historical and recent studies suggest that bone changes may precede changes in other joint tissues [1,2]. The incidence of OA in humans or animals is associated with abnormal subchondral bone remodelling [3,4] and various studies have shown that increased subchondral bone turnover is accompanied by structural changes of the bone, which include increased thickness of subchondral bone plate (Sbp) and trabecular bone (Tb) [1,5,6], and formation of subchondral bone cysts and peripheral osteophytes [5,7,8,9], as evidenced by radiographic and morphometric analysis of the osteoarthritic joint. Other studies have demonstrated that there is an increase bone mineral content (BMC) [10] and bone mineral density (BMD) [11,12] in OA. In our recent studies, we have investigated the sequence and the relationship between bone remodelling and cartilage degradation during development of OA in two spontaneous animal models [13,14]. Our data show that subchondral bone thickening precedes cartilage degradation and chondrocyte apoptosis. All of the above studies support the importance of subchondral bone changes in the pathogenesis of OA and suggest that both cartilage degradation and synovial inflammation may be secondary to subchondral bone changes in OA.

The subchondral bone consists of two compartments; the bone plate, which is cortical bone, and underlying trabecular bone. The two regions of subchondral bone are very different both physiologically and mechanically, and, therefore, it is important to distinguish between the two regions in any investigation of bone changes during development and progression of OA. There are only a few studies that have investigated the differences in bone remodelling between subchondral bone plate (Sbp) and the trabecular bone (Tb) and how they change during initiation and progression of OA. Accordingly, the aim of the current study is to determine the pattern, sequence and the relationship between Sbp and Tb remodelling, during development of OA in two spontaneous animal models, and to test the hypothesis that bone changes in the OA prone DH guinea pig which develop OA around 12 weeks will be more pronounced and rapid compared to the BS2 strain which has a delayed development of OA around 24 weeks of age.

## 2. Results

### 2.1. General Observation

**Figure 1:**
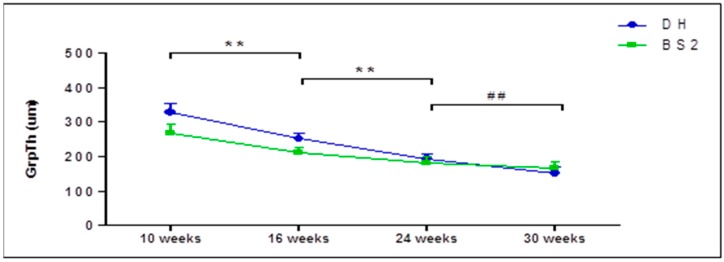
Growth plate thickness (GrpTh) of the proximal tibial epiphysis of DH and BS2 at four different time points. GrpTh was compared between the successive time points (Kruskal-Wallis test). A significant difference of *p* ≤ 0.01 in both strains was denoted as **, while *p* ≤ 0.01 only in DH was denoted as ^##^. Error bars represent the 95% CI.

In both strains, the growth plate was still open at the beginning of the study and had not completely closed by 30 weeks of age. Both strains had a similar closure rate of the growth plate (both in the medial or lateral side) prior to 24 weeks, but after that it was only in DH that the thickness of the growth plate continued to decrease significantly (Figure 1).

**Figure 2:**
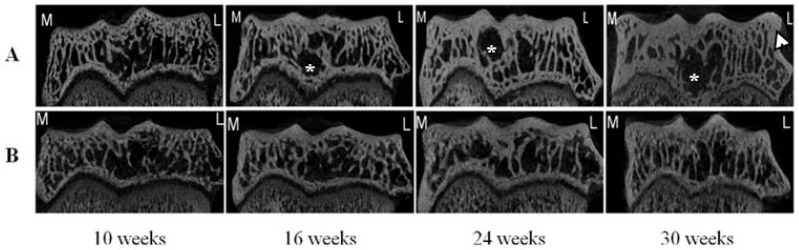
Frontal sections of micro-CT images of right tibial epiphysis of DH (**A**) and BS2 (**B**) at four time points. The images show the SbpTh and Tb morphometry changes on the medial (M) and the lateral sides (L) of the tibial epiphysis. Subchondral bone cyst (*) and osteophyte (arrow head) are seen in some of the images.

Typical micro-CT images of right tibial epiphysis (frontal section) of DH and BS2 at four time points are shown in Figure 2A,B, respectively. SbpTh increased in both strains during the study period and the Tb changes are more apparent in the DH than in BS2 strain. Subchondral bone cysts (*) often appeared underneath the ligament insertion site of the DH, as early as 16 weeks of age. By 30 weeks, osteophytes (Figure 2A, arrow head) were commonly seen at the margin between the articular cartilage (AC) and subchondral bone.

### 2.2. Sb Thickness and Density Changes in OA

The data presented in Table 1 shows that in both, DH and BS2, the SbpTh and TbTh increased in a time dependent manner and were more pronounced in the DH than the BS2, and on the medial than the lateral side. In the medial side, a significant increase of SbpTh was only found in DH between 10 and 16 weeks of age (*p* < 0.01). Little difference was seen between the BMD of Sbp and Tb either by breed or compartment (Table 1). Across the time points, the Sbp BMD of BS2 showed a small gradual increase over the study period. However, in the DH strain an initial increase in BMD of Sbp was followed by a decrease, and this initial increase between 10 and 16 weeks was only significant in the medial (*p* < 0.01), but not in the lateral side.

### 2.3. Rate of Sb Thickness Changes in OA

**Figure 3:**
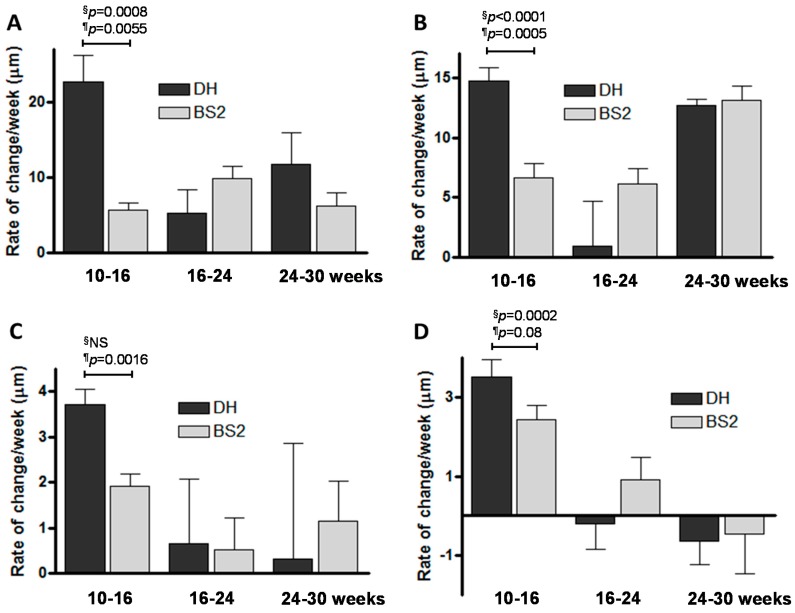
Rate of change in subchondral bone thickness of DH and BS2 guinea pigs for intervals 10–16, 16–24 and 24–30 weeks. (**A**) Medial SbpTh; (**B**) lateral SbpTh; (**C**) medial TbTh and (**D**) lateral TbTh. The rate of change in subchondral bone thickness between strains over time intervals were ranked and compared using 1-way ANOVA ^§^ and Student *t*-test ^¶^. The *p*-values shown are for the earliest time point only.

The rate of change in SbpTh between 10 and 16 weeks is greater in both the medial and lateral compartments for the DH strain (22.7, 14.75 µm/week, respectively) compared with the BS2 (5.63, 6.67 µm/week, respectively; Figure 3A,B). Similarly, the 10–16 week rate of change in TbTh is greater in both medial and lateral compartments for the DH (3.72, 3.52 µm/week, respectively) compared with the BS2 (1.92, 2.43 µm/week, respectively; Figure 3C,D).

### 2.4. Rate of Sb BMD Changes in OA

**Figure 4:**
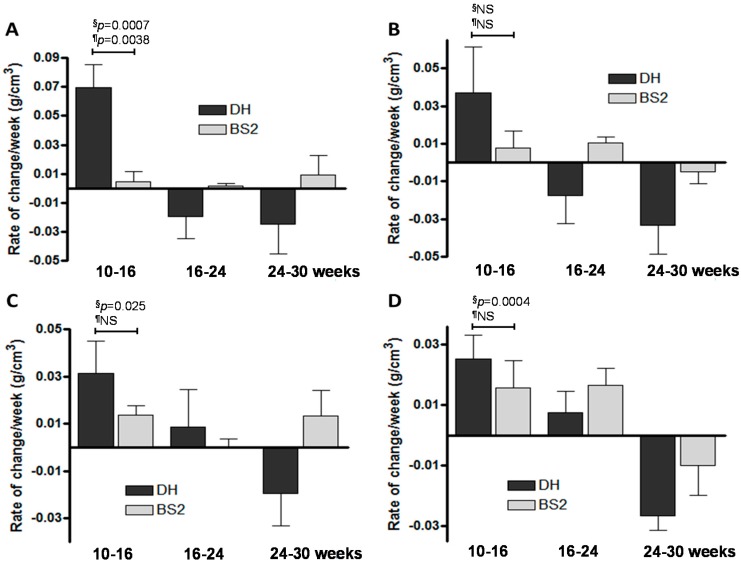
Rate of change in subchondral BMD of DH and BS2 guinea pigs for intervals 10–16, 16–24 and 24–30 weeks. (**A**) Medial Sbp BMD; (**B**) lateral Sbp BMD; (**C**) medial Tb BMD and (**D**) lateral Tb BMD. The rate of change in subchondral bone BMD between strains over time intervals were ranked and compared using 1 way ANOVA ^§^ and Student *t*-test ^¶^. The *p*-values shown are for the earliest time point only.

The 10–16 week rate of change in Sbp BMD is greater in both the medial and lateral compartments for the DH strain (0.0698, 0.0372 g/cm^3^/week, respectively) compared with the BS2 (0.00457, 0.00772 g/cm^3^/week, respectively; Figure 4A,B). In a similar pattern, the 10–16 week rate of change in Tb BMD is greater in both medial and lateral compartments for the DH (0.0314, 0.0253 g/cm^3^/week respectively) compared with the BS2 (0.0135, 0.0157 g/cm^3^/week, respectively; Figure 4C,D).

## 3. Discussion

The data presented here provide unique insight into the early pathogenesis of OA and challenges the conventional theories of initiation and progression of OA. In this study, we have focused on the early changes (10–16 weeks) in subchondral bone that may account for initiation of OA pathology, particularly in the DH guinea pig. Our data show that early subchondral bone parameters change more rapidly in DH (which develops OA early) than the BS2 control strain. The potential link with early OA pathology was corroborated by the lateral compartment, which is less prone to OA in this model, having greater similarity between the strains for the parameters measured. Since, both in animal models and in human, OA develops first in the medial tibiofemoral compartment due to the larger forces experienced in the medial side than the lateral side [6,13,15,16], the early and rapid increase in Sbp thickness and BMD may reflect increased bone remodelling associated with initiation of OA in the medial side, particularly in the DH. The greater volatility in subchondral bone parameters particularly in the medial compartment for the more disease prone strain at a time point which precedes microscopic disease or chondrocyte apoptosis, suggests involvement in the early pathogenesis of OA. There were little or no BMD changes in medial side of the Sbp in BS2. Moreover, the highest rate of BMD increase was also observed in the medial side of Sbp in DH during the earliest time interval. Across all time points, parameters analysed, and compartment measured, there was greater variability in the DH strain than the BS2. The involvement of subchondral bone changes in OA has long been recognised in human disease [17], in induced OA [18], and in spontaneous animal models of OA [11,12,19,20]. This is the first study to identify variability in subchondral bone parameters as a risk factor in OA.

During the early time point (10–16 weeks) the rate of Sbp thickening was higher in the medial than lateral side in the OA prone DH stain but this was less apparent in the BS2. Our observation that there was little medial/lateral differences in TbTh suggests that the Sbp plays a greater role in disease development in early OA. The early time frame of the differences observed here between the disease prone and control strains has been identified in studies of cruciate ligament laxity [21]. It may be that joint instability and rapid changes in joint loading due to ligament laxity is driving volatility in the subchondral bone, which then leads to pathological changes in the cartilage.

When comparing the disease prone DH strain with the control BS2 strain, it was clear that the magnitude of differences at the earliest interval (10–16 weeks) were greater in the subchondral bone plate than the trabecular bone for both thickness and BMD. This was apparent in both medial and lateral compartments. For instance for the medial compartment the differences (DH vs. BS2) were 4-fold and 15-fold for Sbp thickness and BMD respectively, whereas for Tb the differences were 1.9-fold and 2.3-fold, respectively. A previous study investigating both static and dynamic bone metabolism reported that in ovariectomised Cynomolgus monkeys various indices of bone turnover was higher in the subchondral bone compared to cancelous bone [22]. Our observation is, therefore, consistent with previous data and suggests that volatility in the subchondral bone plate is greater than in the trabecular bone, and that this may be a more significant element in early pathology. If this hypotheses is correct, then the search for potential disease modifying drugs for OA targeting bones will have to adopt a more specific approach and aim to normalize bone remodeling in the subchondral bone plate.

It was noticed that SbpTh continued to increase markedly throughout the study period, and that increase in TbTh was less apparent. On the other hand, the Sbp and Tb BMD increased considerably at the earliest time interval in the DH guinea pigs, but then declined to original levels by 30 weeks. This may be due to an increase in porosity in subchondral bone in response to stress shielding. There was little increase or subsequent decrease in subchondral bone BMD in the control strain.

Another important observation reported here is the presence of Sb cysts near the cruciate ligament insertion site as early as 16 weeks in the DH strain. This is consistent with other reports of presence of Sb cysts in DH [5,23,24]. Sb cysts have also been observed in other areas such as the central region of the medial tibial compartment [5] and in the femoral condyles [25,26]. Despite conflicting evidence about when the subchondral cyst first appears in DH [24,25,26], it is commonly seen in the human knee joint with advanced OA [27]. A longitudinal study of human knee OA demonstrated that the Sb cyst may progress in size over time, and are positively associated with AC loss and are predictive of knee surgery [27]. Interestingly, marrow lesion/odema identified and reported by various MRI studies are thought to be early features of OA and correlate with joint pain [28,29], Sb cysts are also known to correlate with bone marrow lesion/odema [30]. Accordingly, our data showing presence of Sb cysts early in the disease process in the DH suggest that Sb cyst may be associated with the early development of OA in this animal model. This is an unexpected and interesting observation and clearly the role of bone cyst in early disease process requires further investigation.

The micro-CT images show that the growth plates of DH and BS2 had not completely closed by the end of the study period. This finding is in agreement with a previous study of DH, which showed complete fusion of the proximal tibia growth plate after one year of age [24]. Moreover, the present study also found that there is a significant decrease of growth plate thickness (GrpTh) in DH up to 30 weeks, which contradicts the findings of Watson et al. [25]. In their study, they found that the longitudinal growth in the DH tibia was not observed after 12 weeks of age [25]. The reason for these discrepancies is not clear. However, Summer-Smith et al. suggested that the cessation of longitudinal bone growth may precede radiographic signs of the growth plate closure [31].

A possible problem with investigating Sb changes prior to the closure of the growth plate is that the bone changes may be subject to the effects of growth. In the present study, both DH and BS2 had a similar closure rate of the growth plate and body weight gain for the first 24 weeks of age [13], yet significantly greater volatility in Sbp thickness and BMD were observed in the medial side at the early time point in the DH compared to BS2. Therefore, the identified changes in the Sb are more likely to be due to the OA pathology than differences in growth patterns. The sample size (*n* = 6) at each time point in our study is rather small however, in previous studies significant relationships were seen with group sizes of 4 at each time point [11], and power calculations show that a sample size of 5 is sufficient to give a 90% chance of detecting a difference between groups with a significance level of 0.05 or less. Other possible limitations of the study may be over or underestimation of the rate of subchondral bone changes as rates were determined in ex vivo in which the data were compared between the time-points based on the group’s mean rather than individual animal’s reading.

## 4. Materials and Methods

### 4.1. Animals

Forty eight, 8 weeks old male guinea pigs (24 BS2 and 24 DH) were used in the study. BS2 guinea pigs were inbred at the Animal Services Unit of University of Bristol and used as growth-matched controls for DH guinea pigs. The DH guinea pigs were purchased from Harlan (Harlan Laboratories Ltd., Bicester, UK) which develop OA around 12 weeks. The animals were housed individually/paired in 23 cm × 52 cm × 72 cm cages, maintained in a 12 h-lighting/22 °C controlled room, and fed with standard guinea pig chow (Harlan Teklad, Bicester, UK) and water (contained 1–2 mg/mL of vitamin C) ad libitum. This experimental study and animal handling protocols were approved by the research ethics committee, University of Bristol (UB/10/024, July, 2010).

### 4.2. Micro-Computed Tomography Scanning (Micro-CT)

Six animals from each strain were euthanized at 10, 16, 24 and 30 weeks of age by an overdose of euthatal via intraperitoneal injection. The right knee joints were dislocated and the tibias were cleaned of soft tissues prior to bone scanning. The proximal part of the tibia was scanned using a micro-CT scanner (Bruker SkyScan 1172, Kontich, Belgium) at 180° rotation with a resolution of 14.7 µm in AP position. The voltage of the X-ray tube was set to 65 kV and the current was 153 µA with a 0.5 mm aluminium filter. The image reconstruction, using a modified Feldkamp algorithm, was undertaken in the software program NRecon (Version 1.6.1.5, Skyscan, Kontich, Belgium) and images were saved in bmp format. The data were analysed using Bruker SkyScan CT-Analyser software (Version 1.9.2.5, Skyscan).

### 4.3. Growth Plate (GpTh) and Subchondral Bone Plate (SbpTh) Thickness

A set of reconstructed images of each sample was opened in CTAn program. By using the multiple re-slice model option, three non-consecutive frontal sections were made in the middle of the tibial plateau. A standard grid was superimposed on each section so that the SbpTh were measured at the same regions across the samples. With the grid as a guide, vertical lines were drawn from the osteochondral junction down to marrow space on the medial/lateral side of the tibial plateau. Using the same frontal sections, the thickness of the medial and lateral side of the growth plate (GpTh) was measured.

### 4.4. Trabecular Bone Thickness (TbTh)

A cylindrical volume of interest (VOI) (diameter: 2 mm; height: 0.36 mm) was placed in the middle of the condyles on each side of the tibial epiphysis. Anatomical landmarks were used as a guide to ensure that the volume of interest (VOI) was positioned in such a way that the subchondral bone plate (Sbp) and the growth plate were excluded. A number of Tb structural parameters were selected for a 3D analysis of the VOI, which include the trabecular bone thickness (TbTh).

### 4.5. BMD Measurement Using a Micro-CT

Two densitometric phantoms of known BMD (0.25 and 0.75 g/cm^3^ of calcium hydroxyapatite) were scanned in similar condition to the tibia. Attenuation coefficient value of each phantom was recorded and used for BMD calibration process. The BMD of Sbp and Tb was measured in the middle of medial/lateral tibial plateau (VOI: diameter: 2 mm; height: 0.15 mm) and in the same region as described in the Tb morphometry analysis, respectively.

### 4.6. Rate of Changes in the Bone Parameters

The rate of change was calculated as the difference between two the time points of each bone parameter divided by the difference between the corresponding ages in weeks.

### 4.7. Statistical Analysis

The data were presented as mean ± SD or ± SEM. Comparisons were made between strains over time intervals 10–16, 16–24 and 24–30 weeks, with rate of change calculated. Statistical analysis was performed using Graphpad Prism software (Graphpad Software, San Diego, CA, USA). Rates of change were ranked, and ANOVA with Bonferroni post-hoc test performed. Student *t*-test was also performed between the groups.

## 5. Conclusions

In summary, our data suggest that rapid and early changes occur in the subchondral bone plate and trabecular bone of the OA prone DH strain compared with the BS2. These changes are most pronounced in the medial tibiofemoral compartment, where disease is seen to develop first. The more pronounced volatility in Sbp than Tb parameters suggests that it is bone plate changes in the medial compartment of the DH strain that are driving pathology. The magnitude and timing of the very early changes (10–16 weeks) are indicative that subchondral bone imbalance may be associated with initiation of OA rather than its progression. Finally, there is evidence that Sb cysts may also be associated with early pathology and development of OA since cysts were observed as early as 16 weeks in the OA prone DH strain.

## Figures and Tables

**Table 1:** The mean of subchondral bone thickness and density in DH and BS2 during the 30 weeks study period.

Medial/Lateral Side	Bone Parameters	10 Weeks				16 Weeks				24 Weeks				30 Weeks			
		DH		BS2		DH		BS2		DH		BS2		DH		BS2	
		Mean	SD	Mean	SD	Mean	SD	Mean	SD	Mean	SD	Mean	SD	Mean	SD	Mean	SD
Medial	SbpTh (µm)	390.4 ^‡^	77.1	411.6 *	58.1	526.6 *^,†^	29.0	445.4 *	44.1	569.3 *	74.9	524.1 *	55.0	639.8	61.8	561.2	64.0
	TbTh (µm)	119.4 *	3.3	112.9 *	6.3	141.7 ^†^	4.5	124.5	7.9	146.9	30.7	128.7	8.0	148.8	19.5	131.4	9.6
	Sbp BMD (g/cm^3^)	1.3 ^‡^	0.2	1.3	0.1	1.7 *^,†^	0.2	1.3	0.1	1.5 *	0.3	1.4	0.1	1.4 *	0.1	1.4	0.2
	Tb BMD (g/cm^3^)	0.3	0.1	0.4	0.1	0.5	0.2	0.5	0.0	0.6	0.2	0.5	0.0	0.5 *^,‡^	0.1	0.5	0.1
Lateral	SbpTh (µm)	365.3	29.5	324.3	52.0	453.8 ^†^	41.1	360.9	41.3	507.2	57.0	462.0 ^‡^	41.2	512.7	104.0	540.6 ^‡^	50.9
	TbTh (µm)	108.2 ^‡^	3.8	106.1 ^‡^	4.8	129.2	9.2	120.7	6.3	127.7	11.7	128.0	9.2	123.9	9.4	125.4	8.4
	Sbp BMD (g/cm^3^)	1.2	0.2	1.3	0.1	1.5	0.2	1.3	0.1	1.3	0.2	1.4	0.1	1.1 ^†^	0.1	1.4	0.1
	Tb BMD (g/cm^3^)	0.3	0.1	0.3 ^‡^	0.1	0.4	0.1	0.4	0.0	0.5	0.1	0.5	0.1	0.3 ^†^	0.1	0.5	0.1

* A significant differerent between the sides of the same strain and time point (*p* < 0.05); † A significant differerent between the strains of the same side and time point (*p* < 0.05); ‡ A significant differerent between the adjacent time points of the same strain and side (*p* < 0.05).
